# Time-sensitive testing pressures and COVID-19 outcomes: are socioeconomic inequalities over the first year of the pandemic explained by selection bias?

**DOI:** 10.1186/s12889-023-16767-5

**Published:** 2023-09-26

**Authors:** Alice R Carter, Gemma L Clayton, M Carolina Borges, Laura D Howe, Rachael A Hughes, George Davey Smith, Deborah A Lawlor, Kate Tilling, Gareth J Griffith

**Affiliations:** 1 MRC Integrative Epidemiology Unit, University of Bristol, Oakfield House, Oakfield Grove, Bristol, BS8 2BN UK; 2 Population Health Sciences, Bristol Medical School, University of Bristol, Oakfield House, Oakfield Grove, Bristol, BS8 2BN UK

**Keywords:** Health inequalities, Selection bias, COVID-19, Socioeconomic position, Linkage studies

## Abstract

**Background:**

There are many ways in which selection bias might impact COVID-19 research. Here we focus on selection for receiving a polymerase-chain-reaction (PCR) SARS-CoV-2 test and how known changes to selection pressures over time may bias research into COVID-19 infection.

**Methods:**

Using UK Biobank (N = 420,231; 55% female; mean age = 66.8 [SD = 8·11]) we estimate the association between socio-economic position (SEP) and (i) being tested for SARS-CoV-2 infection versus not being tested (ii) testing positive for SARS-CoV-2 infection versus testing negative and (iii) testing negative for SARS-CoV-2 infection versus not being tested. We construct four distinct time-periods between March 2020 and March 2021, representing distinct periods of testing pressures and lockdown restrictions and specify both time-stratified and combined models for each outcome. We explore potential selection bias by examining associations with positive and negative control exposures.

**Results:**

The association between more disadvantaged SEP and receiving a SARS-CoV-2 test attenuated over time. Compared to individuals with a degree, individuals whose highest educational qualification was a GCSE or equivalent had an OR of 1·27 (95% CI: 1·18 to 1·37) in March-May 2020 and 1·13 (95% CI: 1.·10 to 1·16) in January-March 2021. The magnitude of the association between educational attainment and testing positive for SARS-CoV-2 infection increased over the same period. For the equivalent comparison, the OR for testing positive increased from 1·25 (95% CI: 1·04 to 1·47), to 1·69 (95% CI: 1·55 to 1·83). We found little evidence of an association between control exposures, and any considered outcome.

**Conclusions:**

The association between SEP and SARS-CoV-2 testing changed over time, highlighting the potential of time-specific selection pressures to bias analyses of COVID-19. Positive and negative control analyses suggest that changes in the association between SEP and SARS-CoV-2 infection over time likely reflect true increases in socioeconomic inequalities.

**Supplementary Information:**

The online version contains supplementary material available at 10.1186/s12889-023-16767-5.

## Background

Numerous studies have sought to identify characteristics associated with elevated risk of SARS-CoV-2 infection and COVID-19 disease, using a range of existing cohorts [1], new data collection such as surveillance sampling [2] and the analysis of routinely collected electronic healthcare records [3, 4]. Such studies have been informative in understanding risk factor associations for SARS-CoV-2 infection and severe COVID-19 disease [5].

Over the course of the pandemic, associations between many individual and contextual risk factors, including socioeconomic position (SEP), and COVID-19 outcomes changed [6–10]. This is to be expected given that SARS-CoV-2 infection results in immunity. Were all individuals at equal risk of infection given their exposure at the start of the pandemic, we might expect those with prior exposure to be less susceptible to reinfection and subsequent disease as the pandemic progressed [11].

However, ascribing changes in risk factor patterning of COVID-19 outcomes to an individual-level aetiological process of acquired infection or subsequent immunity, requires two strong assumptions. Firstly, we must assume that the relationship between the risk factor and the likelihood of exposure to SARS-CoV-2 has not changed over the study period [12]. If, for instance, there were a period where more disadvantaged socio-economic groups were more likely to be exposed to SARS-CoV-2, then we might see a strengthening of the association between more disadvantaged SEP and severe COVID-19 due to this increased risk of exposure.

A less well-appreciated assumption, however, is that we must assume the relationship between the risk factor of interest (e.g., SEP) and being included in the study sample has not changed over time. Where studies use a random or representative sample of the target population and COVID-19 status is ascertained through non-symptom-based testing analyses should not be biased by sample selection however this is rarely the case [8]. For instance, many analyses make use of existing cohort studies and/or rely on linkage to symptom-based national testing programmes (e.g., the UK Biobank was linked to public health polymerase chain reaction (PCR) testing programmes). In this case, if studies look for changes in associations amongst those tested, and access to testing has systematically changed over time for different groups, then we might incorrectly assume we are capturing true changes in risk factor profiles when we are truly capturing changes in testing access. This makes establishing causality between risk factors and COVID-19 difficult and identifying whether changes in associations over time are true changes, or changes in testing selection pressures [5, 13, 14].

This mostly untested assumption of equal or random access to testing is of critical relevance to social theorists and epidemiologists interested in COVID-19. Proxies for SEP have been shown to be strongly associated with access to COVID-19 testing across many but not all contexts [15–18].

### Socioeconomic position (SEP) and COVID-19 in the United Kingdom (UK) context

Like many high-income countries, in the UK, more privileged SEP was observed to be protective against SARS-CoV-2 infection and severe COVID-19 disease early in the pandemic [15, 19, 20]. This could be due to socioeconomic differences in the aetiology of exposure, infection, or disease progression. For instance, more privileged SEP may be associated with reduced likelihood of SARS-CoV-2 exposure due to being able to work from home or lower likelihood of overcrowding [21]. Similarly, more privileged SEP may associated with reduced likelihood of SARS-CoV-2 infection given exposure due to differential immune function [22, 23]. Finally, greater socio-economic privilege may be associated with reduced likelihood of severe COVID-19 outcomes given SARS-CoV-2 infection due to reduced likelihood of comorbidities associated with severe COVID-19 [24].

As the pandemic progressed, the strength, and in some instances the direction, of the association between SEP and SARS-CoV-2 infection and/or severe COVID-19 disease association changed. However, different sources of data for analysis often resulted in different results [6–10].

In England using the Office for National Statistics (ONS) index of multiple deprivation (IMD) data and COVID-19 testing data from Public Health England (PHE), it was found that the association between more disadvantaged area-level SEP and worse COVID-19 outcomes reversed towards the end of 2020, with higher levels of neighbourhood deprivation appearing protective against COVID-19 infection and mortality [6, 9]. This was a period when locally targeted lockdown restrictions were implemented, with lower area-level deprivation becoming risk-inducing for infection and severity for several months before reverting [6, 9, 25, 26].

Results from the ONS COVID-19 Infection Survey (non-symptom-based surveillance data) found little evidence of an effect of area-level deprivation on SARS-CoV-2 positivity from 19th July to 1st August (notably, study data collection began in July). However, as test positivity increased throughout autumn (September to November), the association between SEP and test positivity increased, in contrast to previous studies suggesting a decline in SEP effects [8].

Identifying the causal process driving this change in the association and explaining differences across data is difficult; it could be due to phylogenetic viral evolution [27], changes in natural or vaccine-acquired immunity, or changes in selection processes in COVID-19 testing or reporting.

As is the focus of this paper, evidence shows that in the UK context, testing access was profoundly non-random, and differentially non-random at different points of the UK pandemic, according to testing guidance and lockdown restriction [5, 15]. If testing itself were unrelated to risk factors of interest, or if receiving and accurately reporting a COVID-19 test result were randomly distributed in our target population then conditioning analyses on testing would not present an issue. Where a risk factor of interest is associated with obtaining a test, subsequent analyses that implicitly control for being tested (i.e., comparing tested cases and controls or assuming all untested individuals are suitable controls), can be biased through non-random selection and differential misclassification [14]. However, in the UK COVID-19 testing has been linked to SEP, through a variety of mechanisms such as direct financial barriers to commercial (Pillar 2) testing [28] or indirectly disincentivised testing through inadequate provision of sick leave for precarious workers unable to work from home [2, 21].

Routine surveillance studies with random, population-based testing is the ideal situation for identifying risk factors for SARS-CoV-2 infection and severe COVID-19 disease. Unfortunately, such data are not always accessible or may not have collected information on all variables required for analyses. Therefore, many analyses rely on the use of existing cohort studies with recently collected data on COVID-19 outcomes, potentially using non-representative testing data. Major benefits of these studies include that they have a wealth of pre-pandemic data, have an engaged population of participants for COVID-19 follow-up studies, and have existing access procedures for researchers. However, cohort studies are typically affected by non-random sampling in both COVID-19 data (e.g., ascertaining COVID-19 status through self-report measures, or linkage to symptom-based COVID-19 testing) [29] and non-COVID-19 data [30] (i.e., variables measured pre-pandemic). In this paper, we focus on selection bias in COVID-19 data.

Whilst in general, researchers want to include maximal data to optimise statistical power, where data combines multiple time periods with changing selection pressures (i.e., factors determining, or associated with selection in the study sample), association estimates, which are aggregated across time periods, may not be transportable, because of differential selection bias. This is particularly relevant in the UK context, where all COVID-19 restrictions, including mass testing capacity, were removed in March 2022 [31], this lack of transportability is likely increasingly relevant, as test recipients become more strongly selected. As such, ruling out whether differential testing pressures may drive changes in risk factor associations is of considerable benefit to public health researchers and epidemiologists in understanding changes in COVID-19 risk.

### Study description

This paper contributes to the literature by exploring 3 pertinent questions relating to the impact of selection bias on the association between SEP and SARS-CoV-2 infection using the UK Biobank cohort data and linkage to PHE PCR testing data. Firstly, taking a hypothesised relationship between SEP and SARS-CoV-2 testing over the first year of the UK pandemic, we investigate whether changes to testing access and lockdown restrictions could introduce time-specific selection bias into analyses of SEP and SARS-CoV-2. Secondly, we consider what happens to risk factor estimates for the association between SEP and SARS-CoV-2 infection when we combine periods of distinct testing pressures in an aggregated analysis over the first year of the pandemic. We include multiple measures of SEP which each capture different aspects of SEP which may be important for pandemic resilience. We also use hypothesised positive (ABO blood group) and negative (hair colour) control exposures [32, 33] to further explore the contribution of sample selection.

## Methods

### UK Biobank

UK Biobank is a population-based cohort study which recruited UK adults from 2006 to 2010 [34]. UK Biobank investigators sent postal invitations to over 9 million individuals, registered within the UK’s National Health Service, aged 40–69, living within approximately 25 miles from one of 22 assessment centres in England, Wales and Scotland. Of those invited, 5.5% of individuals (N = 503 317) opted to take part and were aged 37–73 at recruitment.

At baseline, participants completed touch-screen questionnaires, had measurements and blood samples taken and had face-to-face interviews with study nurses. Some limited follow up assessments have taken place [34]. In response to the COVID-19 pandemic, UK Biobank linked participants with PHE SARS-CoV-2 test data, including information on the date and result of the test. This includes both hospital (pillar 1) and community (pillar 2) testing. Given the time frame of these analyses, only polymerase chain reaction (PCR) laboratory-based tests are included in this data, not lateral flow tests (rapid antigen tests). Although the quality of data linkage has not been assessed, it is assumed to be high, since UK Biobank received fully integrated and automated real-time updates from PHE [1]. This analysis focusses on individuals based in England (see exclusion criteria below), and as such, all data come from PHE. Full details of this linkage have been provided previously [1].

### Distinct testing periods

**Fig. 1:**
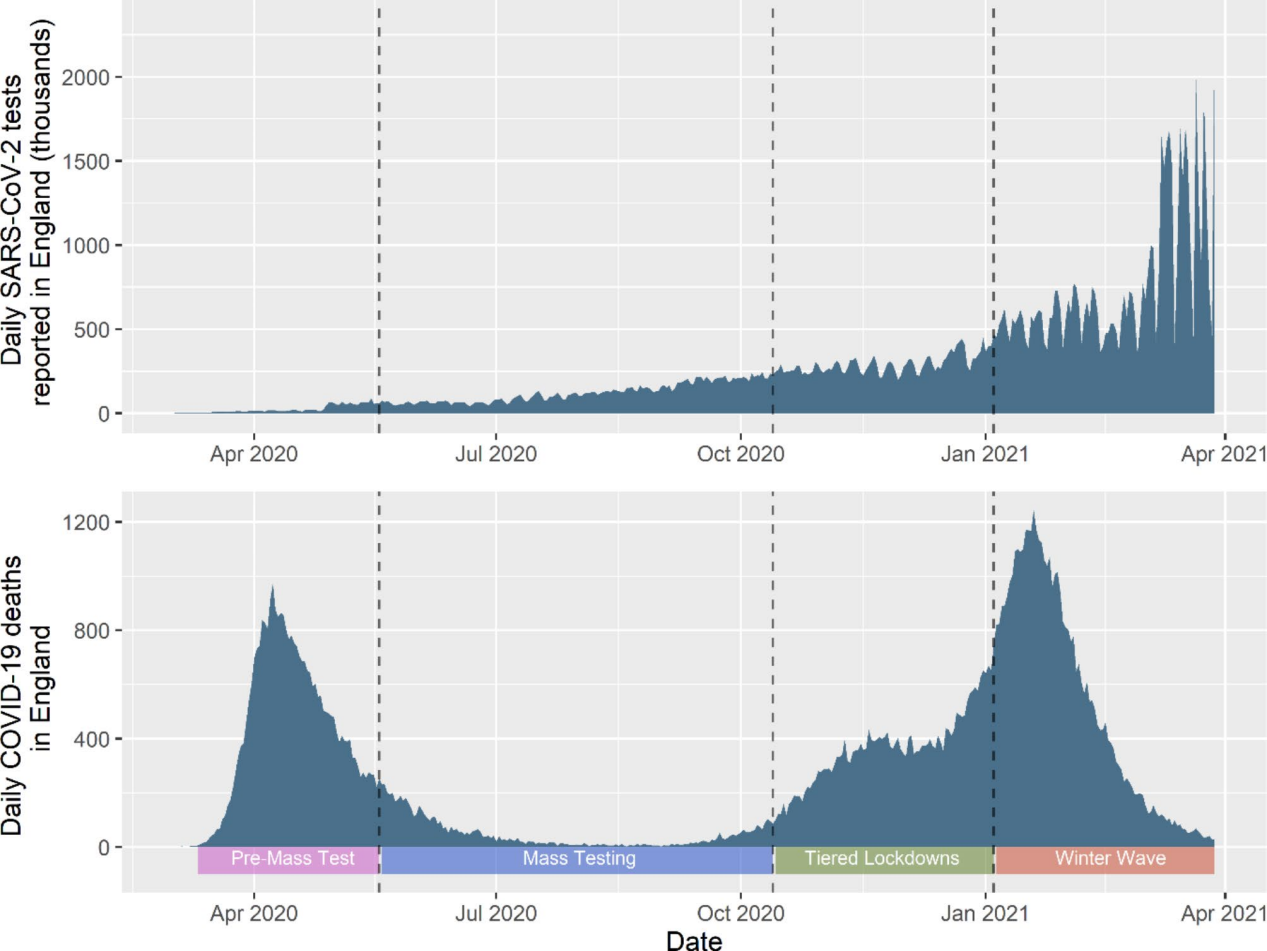
Number of daily reported SARS-CoV-2 tests, as provided by the ONS by date of submission over study period (upper) and number of deaths due to COVID-19 over the study period (lower) [53]. Lower legend, and dotted lines indicate four distinct study periods upon which subsequent analyses are stratified

Four distinct periods of testing during the first 12 months of the pandemic in England were determined *a priori*. These periods were defined based on when selection pressures were anticipated to change due to changing nationwide PCR testing criteria and lockdown restrictions (Fig. 1).

#### Pre-Mass Test period: 11th March 2020 to 18th May 2020

This period starts when the World Health Organisation first declared a pandemic. Little was known about COVID-19, testing capacity was sparse and severe lockdown restrictions were implemented between the 23rd March 2020 and 10th May 2020. The UK Coronavirus Job Retention Scheme (Furlough) began on the 20th March 2020 aiming to avoid redundancies in occupations forced to close during lockdown restrictions and beyond [35]. This scheme remained in effect for the study period and ended on the 30th September 2021 [35].

#### Mass testing period: 19th May 2020 to 13th October 2020

Symptom-based mass testing was introduced on the 19th May 2020 and lockdown restrictions were relaxed during the summer months (although some areas remained in heightened restrictions) [36]. On the 20th September a Test and Trace Support Payment scheme was introduced in England, where low-income individuals in receipt of a government benefit scheme were given a £500 support payment for self-isolation [37]. This remained in effect for the duration of the study period.

#### Tiered lockdown period: 14th October 2020 to 4th January 2021

Tiers-based local restrictions prescribed based on local authority COVID-19 case rates [38, 39] were introduced on the 14th October 2023, implicitly resulting in greater restrictions for more deprived areas. A national “circuit breaker” lockdown was implemented from the 5th November until 2nd December 2020. Local tiered restrictions were then reintroduced, with heightened restrictions for much of the country with some regional variation [38]. This period includes the Christmas of 2020, where some areas of the country were allowed to mix with other households for Christmas Day only.

#### Winter wave period: 5th January 2021 to 28th March 2021

The final period analysed encompasses a full national lockdown which began on the 5th January 2021, until the 28th March 2021, the final day before the “Roadmap out of lockdown” began and the “stay-at-home” order was lifted [38].

### Defining COVID-19 outcomes

Three outcome comparisons were considered (i) tested for SARS-CoV-2 infection versus not tested (to identify factors associated with testing) (ii) tested positive for SARS-CoV-2 infection versus tested negative (to identify risk-factor associations conditioning on testing) and (iii) tested negative for SARS-CoV-2 infection versus not tested. The latter is a negative control outcome indicative of the strength of selection. If testing were random, we would not anticipate difference in risk factor estimates between tested (true) negative participants and untested (assumed) negative participants.

### Defining socioeconomic position

Multiple indicators for SEP were explored in this analysis, representing different life course time points (e.g., highest qualification typically determined in early adulthood and home ownership capturing later adulthood SEP), area- and individual-level measures and indicators of how well individuals may be able to adapt to pandemic restrictions (e.g., income).

All measures of SEP were self-reported at baseline. SEP indicators included were IMD Quintile, home-ownership status, type of accommodation lived in, household income and highest qualification. Specific details for the measurement of each risk factor are provided in Supplementary Table 1.

We hypothesised that the association between SEP and all outcomes would change over time as testing became widely available in the community and that the association would attenuate between SEP and test positivity at time-period 3 when local lockdown restrictions were implemented.

### Defining control exposures

Two control exposures anticipated not to associate with testing were used to evaluate possible bias in our analyses of SEP with COVID-related outcomes. ABO blood type was used as a positive control where we anticipated a time-stable non-zero association with SARS-CoV-2 infection, whilst natural hair colour was used as a negative control where we anticipated a time-stable zero-association with SARS-CoV-2 infection.

An association between ABO blood type and severe COVID-19 disease has been shown previously [40, 41]. Blood type is largely unknown to individuals in the UK, often only being made known to blood donors, a relatively rare subgroup of the population. In England in 2019–2020 there were a reported 801,064 active blood donors out of a population of 67 million [42]. Blood type is genetically determined at conception and unable to be modified by later life exposures. As the association of blood type and COVID-19 was unlikely to be well-known to the public, the effect of blood type on testing, should not exist or change across the time periods. In UK Biobank, blood type is inferred from allele combinations of previously established single nucleotide polymorphisms (rs505922, rs8176746 and rs8176719) in the ABO gene. This variable was derived by Groot et al. and returned to UK Biobank [43]. ABO blood type was available for a maximum of 487 520 participants.

Hair colour before greying was reported by participants at baseline. Although hair colour is known by participants, it is not expected to associate (independent of ethnicity), with either obtaining a test or testing positive for SARS-CoV-2 infection. Therefore, any observed associations can be attributed to selection bias.

### Exclusion criteria

Participants had to be alive at the start of each time-period, i.e., a participant alive at the start of time-period 1 would be included in the first analysis, but if they died before the start of time-period 2, they would not be included in subsequent analyses. To account for previous SARS-CoV-2 infection changing testing behaviour, or natural immunity preventing reinfection, infected individuals were also excluded from the following testing period. For example, if an individual tested positive in time-period 1 they would be excluded from time-period 2 but included again in time-periods 3 and 4. For the overall time period – testing was coded as any test report, individuals were considered a test positive case if they reported at least one positive test result over the entire period, and a test negative case if they reported any negative tests and never tested positive.

**Fig. 2:**
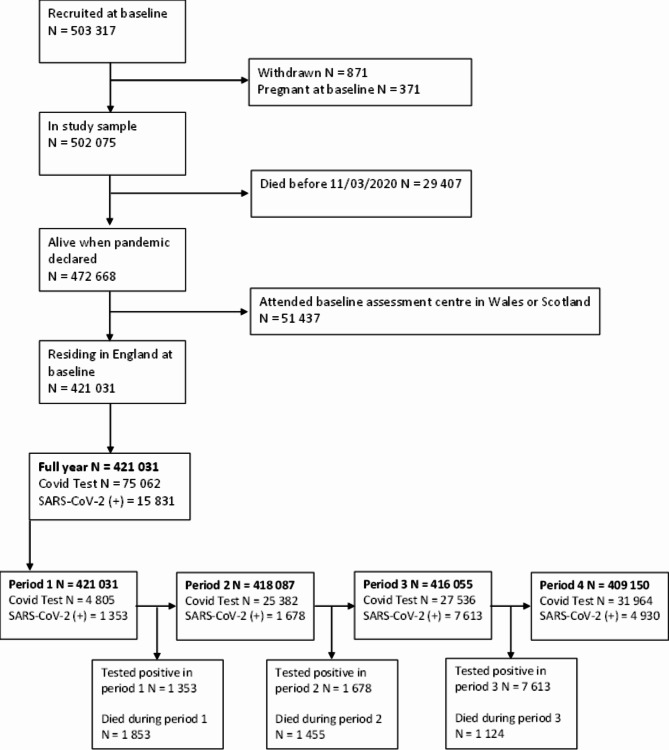
Study flowchart demonstrating inclusion into analyses in UK Biobank. (*Note that an individual could have tested positive for SARs-CoV-2 infection and died from COVID-19)

Testing data were available separately for England, Scotland, and Wales. As each nation set their own restrictions, including dates of lockdowns and capacity for testing, we hypothesised that the temporal-specificity of selection mechanisms would differ between countries. Therefore, UK Biobank participants who attended a baseline assessment centre in Wales or Scotland were excluded. A participant flow diagram is shown in Fig. 2.

### Statistical analyses

#### Associations of SEP, ABO blood type and hair colour with COVID-19 outcomes

Age (at the commencement of study period), sex, and location (UK Biobank baseline assessment centre location and urban/rural household at baseline) adjusted multivariable logistic regression, was used to test the association between each SEP exposure and (i) being tested for SARS-CoV-2 infection (ii) testing positive for SARS-CoV-2 infection (compared with testing negative) and (iii) testing negative for SARS-CoV-2 infection (compared with being untested), stratified by time-period. Age was included as a categorical dummy variable to account for a non-linear associations between age and COVID-19 outcomes. Differences across time-periods were evaluated considering the point estimate and the size of the confidence intervals, including whether these overlapped across periods.

Test positivity was estimated within each time-period for the whole sample and within strata of categorical SEP variables.

Due to ethnicity associating with ABO blood type, hair colour and SEP, analyses of ABO blood type and hair colour were further adjusted for self-reported ethnicity.

For categorical SEP measures the reference category was selected to be indicative of most privileged SEP. For brevity, we present the results for income, education, IMD and ABO blood type in the main text, and all other results in the Supplementary Material.

We explored the association between age and sex independently with testing and infection over time using unadjusted univariable regression.

#### Sensitivity analyses

Analyses considering ABO blood type and hair colour as the exposures were replicated on a sample of principle component analysis (PCA)-selected, ancestrally British participants [44] as a second approach to account for ancestry as a confounder.

Analyses with income as the exposure were replicated excluding participants who reported they were retired at baseline.

## Results

### UK Biobank participant characteristics and missing data

420 231 UK Biobank participants were included in analyses (55% female; mean age 66.75 [standard deviation = 8·11]) (Table 1). Except for income, there was little missing data in exposure and covariate variables (Supplementary Table 1). 4 805 tests (28·16% positive) were conducted in time-period 1, rising to 31 964 at time-period 4 (15·42% positive) (Supplementary Tables 2 and 3).

**Table 1:** UKB participant characteristics (total N = 420 231)

Risk factor	Level of exposure	N (%)
Age		66.75 (8.11)
Sex	Male	188 625 (44.9)
	Female	231 606 (55.1)
Education	Degree or higher	137 578 (33.4)
	Vocational qualifications	115 662 (28.0)
	AS/A level	22 324 (5.4)
	GCSE or less	136 991 (33.2)
Income	Greater than £100,000	19 981 (5.6)
	£52,000-£100,000	73 778 (20.8)
	£31,000–51,599	93 575 (26.3)
	£18,000-£30,999	90 013 (25.3)
	Less than £18,000	78 028 (22.0)
Accommodation type	House of bungalow	379 656 (90.8)
	Flat, maisonette or apartment	36 845 (8.8)
	Mobile or temporary structure (e.g. caravan)	525 (0.1)
	Sheltered accommodation	951 (0.2)
	Care home	57 (0.01)
IMD Quintile	1 (least deprived)	81 593 (20.0)
	2	81 611 (20.0)
	3	81 567 (20.0)
	4	81 596 (20.0)
	5 (most deprived)	81 575 (20.0)
Home ownership	Own outright	215 694 (52.2)
	Own with mortgage	155 193 (37.6)
	Rent - LA/council/housing association	24 031 (5.8)
	Rent - private landlord	13 827 (3.4)
	Shared ownership	1 288 (0.3)
	Live rent free	3 074 (0.7)
Blood type	A	178 251 (43.7)
	B	38 919 (9.5)
	AB	14 812 (3.6)
	O	175 773 (43.1)
Hair colour	Blonde	44 478 (10.6)
	Brown	316 398 (75.6)
	Other	57 455 (13.7)

### Associations with testing for SARS-CoV-2

**Fig. 3:**
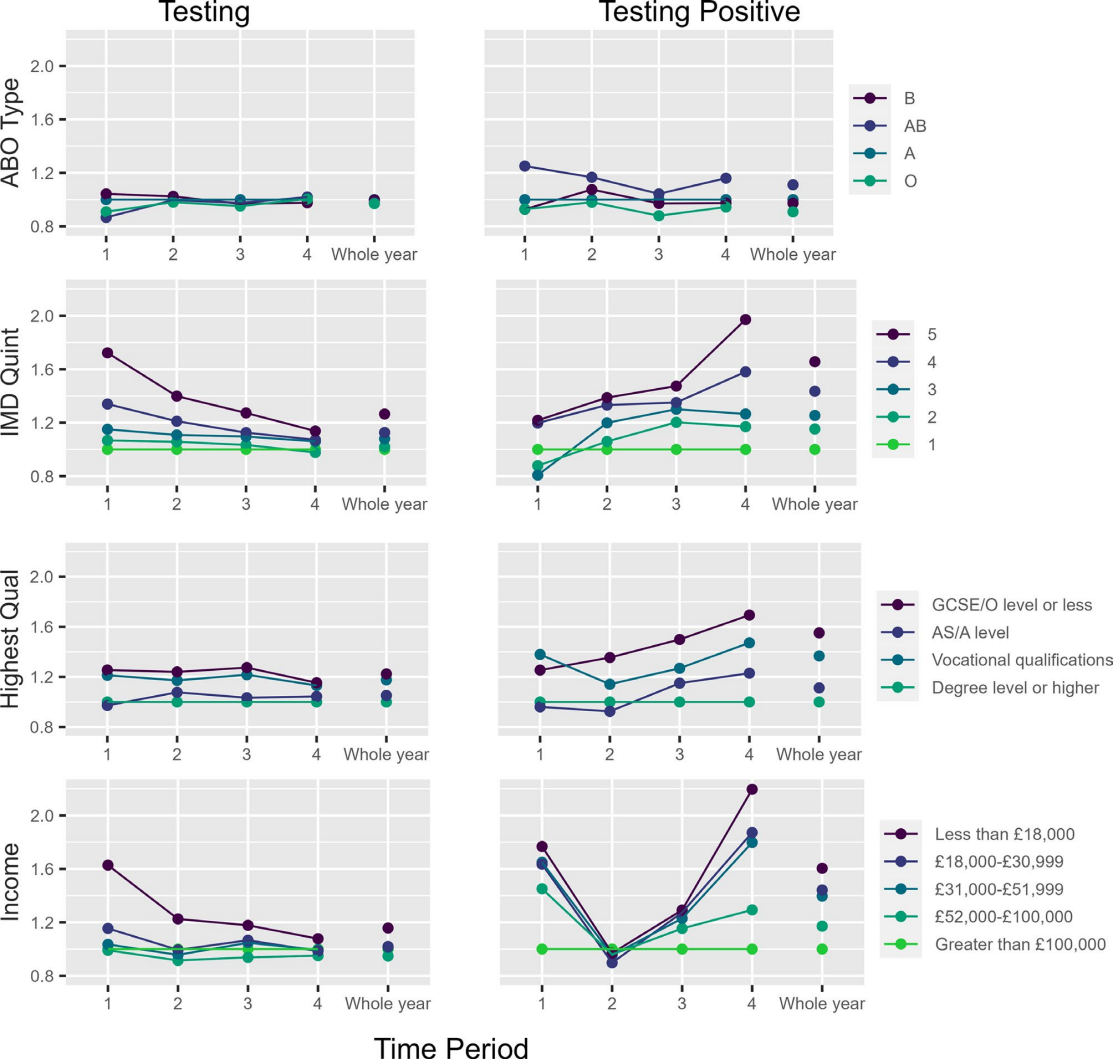
Association of education, income and quintiles of index of multiple deprivation (putative time-varying risk factors) and ABO blood type (putative time-stable control exposure) with SARS-CoV-2 testing and testing positive for SARS-CoV-2 infection

For all SEP exposures, the association between SEP and obtaining a test decreased over time (Fig. 3 and Supplementary Table 4). Considering highest qualification as the exposure, compared with having a degree (or higher), the odds ratio (OR) for the association between having General Certificates of Secondary Education (GCSEs) or less and obtaining a test at time-period 1 was 1·25 (95% confidence interval (CI): 1·16 to 1·35), decreasing to 1·15 (95% CI: 1·12 to 1·19) at time-period 4 (Fig. 3 and Supplementary Table 4).

As hypothesised, both control exposures were not associated with obtaining a test. Considering ABO as a positive control, although the size of the point estimates for each blood type changed across time-periods in most cases the confidence interval overlapped with the null. For example, compared with blood type A, the association between blood type B and obtaining test at time-period 1 was 1·04 (95% CI: 0·94 to 1·15) and 0·98 (95% CI: 0·94 to 1·02) at time-period 4. Comparing blood type O with blood type A, the association with testing at time-period 1 was 0·91 (95% CI: 0·85 to 0·97), and at time 4 was 1·00 (0·98 to 1·03) (Fig. 3 and Supplementary Table 5). Apart from “other” hair colour compared with blonde hair at time-period 1 (OR: 1·19; 95% CI: 1·05 to 1·33), there was little association between hair colour and testing.

### Associations with testing positive for SARS-CoV-2

The association between disadvantaged SEP and testing positive for SARS-CoV-2 infection versus testing negative increased over time (Fig. 3 and Supplementary Table 6). Considering highest qualification as the exposure, compared with having a degree (or higher), the OR for the association between having GCSEs or less and testing positive for SARS-CoV-2 infection at time-period 1 was 1·25 (95% CI: 1·04 to 1·47), increasing to 1·69 (95% CI: 1·55 to 1·83) at time-period 4 (Fig. 3 and Supplementary Table 6).

For all exposures, test positivity varied over time-periods, with the highest test positivity in time-periods 1 and 3 (Supplementary Table 2). Test positivity increased with lower levels of SEP, and the differences increased over time, concordant with the increasing ORs for SEP and SARS-CoV-2 infection (Supplementary Tables 2 and 3).

There was little evidence that either control exposure was associated with testing positive for SARs-CoV-2 infection at any time-period, with associations remaining relatively stable across time-periods (Fig. 3 and Supplementary Table 7).

### Associations with testing negative for SARS-CoV-2

The association between SEP and testing negative for SARS-CoV-2 infection versus not being tested typically decreased over time (Supplementary Table 8). Considering highest qualification as the exposure, compared with having a degree (or higher), the OR for the association between having GCSEs or less and testing negative at time-period 1 was 1·19 (95% CI: 1·08 to 1·29), decreasing to 1·07 (95% CI: 1·04 to 1·10) in time-period 4 (Supplementary Table 8).

There was little evidence that either control exposure (ABO blood type and hair colour) was associated with testing negative for SARS-CoV-2 infection compared with not being tested at any time-period (Supplementary Table 9).

Age and sex were both independently associated with all outcomes (Supplementary Table 10).

### Sensitivity analyses

When restricting to PCA-selected, ancestrally British participants, the association between ABO blood type and hair colour on all outcomes were consistent with main analyses. (Supplementary Table 11).

When excluding participants retired at baseline, the association between income and all outcomes were also concordant with the main analyses (Supplementary Table 12).

## Discussion

In our hypothesis driven SEP analyses, we demonstrate time-sensitive selection processes on receiving a test by SEP. We show the association between SEP (measured by multiple proxies, including IMD, income and education) and SARS-CoV-2 testing declined over the study period. Early in the pandemic (March 2020) more socio-economically disadvantaged individuals were more likely to obtain a test than those with more privileged SEP. However, by March 2021, there was little difference in testing by SEP. In contrast, the association between SEP and SARS-CoV-2 infection strengthened over the course of the pandemic, where more socio-economically disadvantaged participants were increasingly more likely to test positive during the 12 months studied. This could be due to greater exposure to SARS-CoV-2 virus, higher susceptibility to infection or due to differential vaccination rates [45, 46], (where vaccines were approved in December 2020 and were widely rolled out in time-period 4) [47].

We used positive and negative control exposures to investigate potential selection bias [32, 33]. Whilst these analyses do not explicitly tell us whether selection bias is present, they can inform us about whether observed results may be explained by a hypothesised bias. Although there were some variations in the point estimates obtained for ABO blood type and hair colour at different time-periods, the estimates were imprecise with confidence intervals typically spanning the null, suggesting little evidence of selection bias.

We have shown that different time-periods with strong and distinct testing pressures produce a series of results which are not immediately transportable to any specific period. We suggest that researchers using UK Biobank (or other data sources with similar non-random study sampling) to investigate COVID-19 outcomes, consider the temporal testing context, and select included time-periods appropriately. Researchers should use methods to mitigate selection bias where possible, and where not possible use sensitivity analyses, such as control exposures and outcomes to assess the plausibility of selection bias [14, 29]. As universal testing, or representative population-based sampling surveys, come to an end in many places, these considerations remain important.

In support of previous studies, including those from population surveys, we found evidence that risk factor associations between SEP and COVID-19 outcomes (testing and infection) changed over time [7, 26]. During the first 12 months of the pandemic, testing became more widespread and less selective, however, inequalities worsened over this time. Despite a trend towards worsening inequalities, we found an attenuation of the effect of SEP on SARS-CoV-2 infection at time-period 3 when local lockdown restrictions were introduced, supporting previous conclusions from population level data for SEP and SARS-CoV-2 infection [6, 14, 25].

In contrast to previous studies [40, 41], we did not find evidence of an association between ABO blood type and SARS-CoV-2 infection. This could be because previous studies were biased due to non-random sample selection (i.e., conducted early in the pandemic), or they could be due to pre-pandemic selection bias in UK Biobank. Alternatively, differences may be due to the endpoint considered; here we used SARS-CoV-2 infection; however, many previous studies have investigated severe COVID-19 disease or death. Severe-COVID-19 outcomes are also less likely to be subject to selection bias, as severe cases of disease have been well tested from the start of the pandemic.

In this analysis we have considered multiple SEP measures aiming to capture different aspects of SEP and pandemic control measures. We have included IMD as an area-level measure of deprivation and both early (education) and later (income) adulthood measures. We further included measures of SEP which may affect how adaptable individuals are to the pandemic, such as income, where particularly early in the pandemic in the UK it may have been difficult to access a testing site without private transport. Accordant results across the included SEP measures are consistent with SEP encompassing a range of resources relevant to health, representing a fundamental cause of health inequalities, irreducible to a single factor [48]. Many previous studies have only considered area-level SEP [6, 9, 49]. This is often the only available data when using population-based COVID-19 data but limits our understanding of how within-area differences in SEP affect COVID-19 outcomes.

There are limitations of these measures. Individual level data were measured at UK Biobank baseline, up to 14 years before the pandemic began (years 2006–2010). Whilst factors such as education (highest qualification) are unlikely to have changed in adults during this time, other measures, such as income, may have changed, and were these subsequent changes systematically related to socio-economic position or COVID-19 outcomes, then these may bias our reported results. Furthermore, SEP is complex and multi-faceted, such that a single, unidimensional understanding is likely over simplistic or reductive when considering complex causal processes. For our selected exposures, we selected the intuitive “more privileged” SEP responses as a reference category, however this meant for income that the reference category contained relatively few individuals, meaning our income-related estimates are more volatile between time periods. Similarly, where income varies over the lifecourse, for most individuals education will remain static after childhood or early adulthood. Taken together these may explain the weak evidence of incongruent education and income effects in time period 2 (Supplementary Tables 4 and 6). However, by including multiple measures of SEP, we can attempt to disentangle how different factors may have contributed to inequalities in SARS-CoV-2 infection.

In sensitivity analyses, where we removed retired individuals from income analyses, we could only exclude individuals retired at baseline. Whilst this will likely be correlated with income at baseline, we could not unpick how current employment status was associated with COVID-19. Further, we could not examine how associations with occupation, which has been shown to be associated with COVID-19, have changed over time. Whilst some occupation data are available, we do not have access to (i) self-employment status or (ii) The National Statistics Socio-economic classification codes which can be used to proxy SEP.

To explore changes over time, we *a priori* defined four time points where distinct PCR testing pressures exist. We used external information (i.e., government guidance) to define these time points. However, these results may be sensitive to the time periods selected and splitting the data into four (or more) different time points may lead to different results. For instance, over our study period, rapid antigen testing (using lateral flow devices (LFDs)) was not freely available to the public [50]. In the period following October 2020, LFDs started becoming available in limited settings (although available in increasing settings until being made free to the public on 9th April 2021) and any positive lateral flow was required to be confirmed by PCR-testing. The need for PCR test confirmation was relaxed between 27th January 2021 and 29th March 2021 [50], and as such may affect estimates in our final time period. Additionally, despite UK Biobank having over half a million participants, we were limited by the number of COVID-19 cases in some strata, and therefore were unable to investigate more fine-grained time points (e.g., weekly).

UK Biobank is known to be a selected sample itself. Participants are healthier and wealthier than the general population [34], and as such, the point estimates obtained here may not be transportable to other populations, where inequalities at the population level may be underestimated. Additionally, the UK Biobank target population represents a small subset of the UK population (Aged 40–70, living within 25 miles of a test centre), meaning the results are not generalisable across the UK, and bias may be generated from systematic differences in record linkages [51]. Whilst UK Biobank overall has relatively little missing data, some variables (e.g., income) experience high amounts of missingness, which we did not account for. We feel it is plausible to assume that missingness in the reporting of income is missing *not* at random, i.e., the missing data mechanism for income is systematically related to the unobserved income values and therefore complete case analysis is the most appropriate method [52].

## Conclusion

Understanding causes of SARS-CoV-2 infection and subsequent COVID-19 disease requires an understanding of selection pressures leading to inclusion in the study sample (e.g., through testing), and whether any selection generalises across time. We show this assumption does not hold true for UK Biobank, by demonstrating that selection pressures changed across time, with sample selection becoming more representative as widespread, accessible testing became available. Our study presents results across a range of analyses which are consistent with the increasing SEP inequalities in COVID-19 outcomes as the pandemic persisted reflecting a real effect, and not an artefact of non-random testing. This demonstrates the public health and epidemiological importance of readily accessible testing to understand causal risk factors for SARS-CoV-2 infection and COVID-19 disease progression.

### Electronic supplementary material

Below is the link to the electronic supplementary material.

Supplementary Material 1

## Data Availability

The data used in the present study are available to all bona fide researchers for health-related research that is in the public interest, subject to an application process and approval criteria. Study materials are publicly available online at http://www.ukbiobank.ac.uk.

## List of abbreviations

SEP: Socio-Economic Position
UK: United Kingdom
PHE: Public Health England
ONS: Office for National Statistics
IMD: Index of Multiple Deprivation
PCA: Principal Component Analysis
OR: Odds Ratio
CI: Confidence Interval
GCSE: General Certificate of Secondary Education

## Data availability

Code used to produce these analyses are publicly available at https://github.com/alicerosecarter/timevarying_covid_selection.
