# Supplementary material for: Time-sensitive testing pressures and COVID-19 outcomes: are socioeconomic inequalities over the first year of the pandemic explained by selection bias?
Source: BMC Public Health. 2023 Sep 26;23:1863. doi: 10.1186/s12889-023-16767-5 (PMC10521522; doi:10.1186/s12889-023-16767-5)
Supplement: Supplementary file 1 — Supplementary Material 1 [file 12889_2023_16767_MOESM1_ESM.docx]

Table of Contents

[Supplementary Methods 2](#_Toc99111406)

[Measures of socioeconomic position 2](#_Toc99111407)

[IMD 2](#_Toc99111408)

[Household income 2](#_Toc99111409)

[Highest qualification 2](#_Toc99111410)

[Homeownership status at baseline 2](#_Toc99111413)

[Type of accommodation lived in 3](#_Toc99111414)

[Covariates 3](#_Toc99111415)

[Age 3](#_Toc99111416)

[Sex 3](#_Toc99111417)

[Ethnicity 3](#_Toc99111418)

[Genetically determined White British 3](#_Toc99111419)

[Supplementary Tables 4](#_Toc99111420)

[Supplementary Table 1: Proportion of missing data in risk factor variables and subsequent loss of outcome observations 4](#_Toc99111421)

[Supplementary Table 2: Association between test-associated risk factors and SARs-CoV-2 testing 5](#_Toc99111422)

[Supplementary Table 3: Association between test-independent risk factors and SARs-CoV-2 testing 7](#_Toc99111423)

[Supplementary Table 4: Association between test-associated risk factors and testing positive for SARs-CoV-2 infection 8](#_Toc99111424)

[Supplementary Table 5: Test positivity for time-associated risk factors 10](#_Toc99111425)

[Supplementary Table 6: Test positivity for test-independent risk factors 12](#_Toc99111426)

[Supplementary Table 7: Association between test-independent risk factors and testing positive for SARs-CoV-2 infection 13](#_Toc99111427)

[Supplementary Table 8: Association between test-associated risk factors and testing negative for SARs-CoV-2 infection 14](#_Toc99111428)

[Supplementary Table 9: Association between test-independent risk factors and testing negative for SARs-CoV-2 infection 16](#_Toc99111429)

[Supplementary Table 10: Association between test-independent risk factors, ABO blood type and hair colour, with tests for SARs-CoV-2, testing positive for SARs-CoV-2 infection and testing negative for SARs-CoV-2 infection, excluding individuals of non-White British ancestry 17](#_Toc99111430)

[Supplementary Table 11: Association between income and tests for SARs-CoV-2, with tests for SARs-CoV-2, testing positive for SARs-CoV-2 infection and testing negative for SARs-CoV-2 infection, excluding individuals who self-reported being retired at baseline 18](#_Toc99111431)

[Supplementary Table 12: Association between age and sex with for with tests for SARs-CoV-2, testing positive for SARs-CoV-2 infection and testing negative for SARs-CoV-2 infection 19](#_Toc99111432)

# Supplementary Methods

# Measures of socioeconomic position

## IMD

The English Indices of Deprivation (Index of Multiple Deprivation [IMD]) aim to broadly measure local area deprivation. Seven domains of deprivation are included in the score, encompassing crime, education, employment, health, housing, income and living environment scores. For each participant, published IMD scores were matched to their baseline postcode, using the IMD release closest to their date of baseline assessment (2005-2010). Scores are derived separately for England, Wales, and Scotland and scores were not combined in UK Biobank. In analyses, IMD was grouped into equal quintiles, where quintile 1 (least deprived) was considered baseline.

## Household income

Average household income before tax was reported at baseline assessment centres. In analyses, categories of income were used as defined by UK Biobank (greater than £100 000 [baseline], “£52 000 to £100 000”, “£31 000 to £51 599”, “£18 000 to £30 999” and “less than £18 000”). Individuals who answered, “do not know” (N = 19 449) or “prefer not to say” (N = 46 641) were set to missing.

This variable does not account for sources of income, for example, whether income is from active employment or a pension.

## Highest qualification

Participants reported their highest qualification achieved at baseline assessment centres. In analyses, these were grouped into “degree level or higher” (baseline), “vocational qualifications”, “AS/A level” and “GCSE/O level or less”. For anyone responding “prefer not to answer” observations were set to missing.

## Homeownership status at baseline

Participants were asked at baseline “Do you own or rent the accommodation that you live in?” This data was collected from all participants, except those who had previously indicated they were living in sheltered accommodation or a care home. This variable was categorised for analyses into “Own outright” (baseline), “Own with mortgage”, “Rent from local authority, council or housing association”, “Rent privately”, “Shared ownership” and “Live rent free”. Individuals who answered, “do not know” or “prefer not to say” were set to missing.

## Type of accommodation lived in

Participants were asked at baseline “What type of accommodation do you live in?”. Responses were categorised as “House or bungalow” (baseline), “Flat, maisonette or apartment”, “Mobile or temporary structure”, “Sheltered accommodation” and “Care home”. Individuals who answered, “do not know” or “prefer not to say” were set to missing.

# Covariates

## Age

To account for non-linear effects of age on SARS-CoV-2 infection and COVID-19 disease, age at the commencement of the study period (11^th^ March 2020) was calculated and categorised into 5-year age bands. This was then included as a categorical variable. Due to small numbers, individuals aged i) under 40 and ii) over 65 were grouped together.

## Sex

Sex of the participant at baseline was acquired from central registries (the NHS), which could be updated with self-reported gender by the participant.

## Ethnicity

Ethnicity was reported using touch screen questionnaires at baseline assessment centres and categorised into “White”, “Indian”, “Pakistani”, “Bangladeshi”, “Other Asian”, “Black Caribbean”, “Black African”, “Chinese” and “Other”.

## Genetic Principal Component ascertained White British

Individuals categorised as White British were defined by UK Biobank as individuals who self-reported as both “white” and “British” and have similar characteristics (measured by genetic principal components) to typically studied white, British populations.

# Supplementary Tables

Supplementary Table 1: Proportion of missing data in risk factor variables and subsequent loss of outcome observations

| Risk factor | Number of missing observations for risk factor (%) | Number of outcome observations | |
| --- | --- | --- | --- |
|  |  | SARs-CoV-2 tests  (% of cases lost) | SARs-CoV-2 test positive (% of cases lost) |
| Education | 7 676 (1.8) | 73 575 (2.0) | 15 492 (2.1) |
| Income | 64 856 (15.4) | 63 074 (16.0) | 13 418 (15.2) |
| Accommodation | 2 197 (0.5) | 74 612 (0.6) | 15 734 (0.6) |
| IMD | 12 289 (2.9) | 72 969 (2.8) | 15 359 (3.0) |
| Homeownership | 7 124 (1.7) | 73 651 (1.9) | 15 525 (1.9) |
| Hair colour | 1 800 (0.4) | 74 702 (0.5) | 15 741 (0.6) |
| Blood type | 12 476 (3.0) | 72 740 (3.1) | 15 322 (3.2) |

% missing based on a maximum sample size of 420 231

SARs-CoV-2 test total N = 75 062

SARs-CoV-2 test positive total N = 15 831

## Supplementary Table 2: Test positivity for test-associated risk factors

| Risk factor | Level of exposure | Time 1 | | | | Time 2 | | | | Time 3 | | | | Time 4 | | | | |
| --- | --- | --- | --- | --- | --- | --- | --- | --- | --- | --- | --- | --- | --- | --- | --- | --- | --- | --- |
|  |  | Total sample | Tests | Test + | Test positivity % (95% CI) | Total sample | Tests | Test + | Test positivity % (95% CI) | Total sample | Tests | Test + | Test positivity % (95% CI) | Total sample | Tests | Test + | Test positivity % (95% CI) |  |
| Total | | 420 231 | 4 805 | 1 353 | 28.16 (28.15, 28.17) | 418 087 | 25 382 | 1 678 | 6.61 (6.61, 6.61) | 416 055 | 27 536 | 7 613 | 27.65 (27.64, 27.65) | 409 150 | 31 964 | 4 930 | 15.42 (15.42, 15.43) |  |
| **Education** | Degree or higher (REF) | 137 578 | 1337 | 334 | 24.98 (24.96, 25.00) | 137 045 | 7 216 | 402 | 5.57 (5.57, 5.57) | 136 594 | 7 987 | 1 956 | 24.49 (24.48, 24.50) | 134 806 | 9 769 | 1 238 | 12.67 (12.67, 12.68) |  |
|  | Vocational qualifications | 115 662 | 1372 | 430 | 31.34 (31.32, 31.36) | 115 042 | 7 047 | 510 | 7.24 (7.23, 7.24) | 114 493 | 7 840 | 2 304 | 29.39 (29.38, 29.39) | 112 484 | 9 026 | 1 522 | 16.86 (16.86, 16.87) |  |
|  | AS/A level | 22 324 | 208 | 51 | 24.52 (24.48, 24.56) | 22 238 | 1 252 | 70 | 5.59 (5.58, 5.60) | 22 162 | 1 332 | 355 | 26.65 (26.63, 26.67) | 21 820 | 1 646 | 246 | 14.95 (14.93, 14.96) |  |
|  | GCSE or less | 136 991 | 1768 | 501 | 28.34 (28.32, 28.35) | 136 141 | 9 295 | 660 | 7.10 (7.10, 7.10) | 135 226 | 9 817 | 2 849 | 29.02 (29.01, 29.03) | 132 587 | 10 968 | 1 813 | 16.53 (16.52, 16.53) |  |
| **Income** | Greater than £100,000 (REF) | 19 981 | 184 | 38 | 20.65 (20.61, 20.69) | 19 932 | 1 120 | 81 | 7.23 (7.22, 7.24) | 19 860 | 1 291 | 338 | 26.18 (26.16, 26.20) | 19 586 | 1 541 | 181 | 11.75 (11.73, 11.76) |  |
|  | £52,000-£100,000 | 73 778 | 660 | 176 | 26.67 (26.64, 26.69) | 73 530 | 3 698 | 281 | 7.60 (7.59, 7.60) | 73 271 | 4 309 | 1 310 | 30.40 (30.39, 30.41) | 72 153 | 5 306 | 763 | 14.38 (14.37, 14.39) |  |
|  | £31,000-51,599 | 93 575 | 894 | 253 | 28.3 (28.28, 28.32) | 93 211 | 5 050 | 377 | 7.47 (7.46, 7.47) | 92 832 | 6 016 | 1 796 | 29.85 (29.85, 29.86) | 91 268 | 6 996 | 1 187 | 16.97 (16.96, 16.97) |  |
|  | £18,000-£30,999 | 90 013 | 985 | 272 | 27.61 (27.59, 27.63) | 89 571 | 5 376 | 328 | 6.10 (6.10, 6.11) | 89 111 | 5 913 | 1 600 | 27.06 (27.05, 27.07) | 87 606 | 6 755 | 998 | 14.77 (14.77, 14.78) |  |
|  | Less than £18,000 | 78 028 | 1 275 | 362 | 28.39 (28.37, 28.41) | 77 401 | 5 803 | 361 | 6.22 (6.22, 6.23) | 76 866 | 5 645 | 1 476 | 26.15 (26.14, 26.16) | 75 449 | 6 368 | 1 016 | 15.95 (15.95, 15.96) |  |
| **Accommodation type** | House of bungalow (REF) | 379 656 | 4 094 | 1 152 | 28.14 (28.13, 28.15) | 377 822 | 22 418 | 1 534 | 6.84 (6.84, 6.85) | 375 966 | 24 580 | 6 847 | 27.86 (27.85, 27.86) | 369 816 | 28 630 | 4 324 | 15.10 (15.10, 15.11) |  |
|  | Flat, maisonette or apartment | 36 845 | 624 | 180 | 28.85 (28.82, 28.87) | 36 575 | 2 636 | 132 | 5.01 (5.00, 5.01) | 36 423 | 2 659 | 699 | 26.29 (26.28, 26.30) | 3 5736 | 2 972 | 535 | 18.00 (17.99, 18.01) |  |
|  | Mobile or temporary structure (e.g. caravan) | 525 | 7 | 0 | NA | 524 | 37 | 0 | NA | 521 | 37 | 7 | 18.92 (18.83, 19.01) | 512 | 42 | 5 | 11.90 (11.83, 11.97) |  |
|  | Sheltered accommodation | 951 | 44 | 12 | 27.27 (27.18, 27.37) | 932 | 117 | 5 | 4.27 (4.25, 4.30) | 923 | 86 | 20 | 23.26 (23.19, 23.32) | 902 | 118 | 17 | 14.41 (14.36, 14.45) |  |
|  | Care home | 57 | 3 | 0 | NA | 55 | 8 | 1 | 12.50 (12.34, 12.66) | 54 | 10 | 3 | 30.00 (29.80, 30.20) | 52 | 7 | 4 | 57.14 (56.88, 57.4) |  |
| **IMD Quintile** | 1 (least deprived) (REF) | 81 593 | 714 | 192 | 26.89 (26.87, 26.91) | 81 270 | 4 539 | 191 | 4.21 (4.20, 4.21) | 80 971 | 5 000 | 1 015 | 20.30 (20.29, 20.31) | 80 002 | 6 161 | 643 | 10.44 (10.43, 10.44) |  |
|  | 2 | 81 611 | 772 | 190 | 24.61 (24.59, 24.63) | 81 296 | 4 630 | 245 | 5.29 (5.29, 5.30) | 80 962 | 5 025 | 1 278 | 25.43 (25.42, 25.44) | 79 754 | 5 914 | 732 | 12.38 (12.37, 12.38) |  |
|  | 3 | 81 567 | 830 | 195 | 23.49 (23.47, 23.51) | 81 224 | 4 735 | 307 | 6.48 (6.48, 6.49) | 80 816 | 5 242 | 1 449 | 27.64 (27.63, 27.65) | 79 510 | 6 233 | 842 | 13.51 (13.50, 13.51) |  |
|  | 4 | 81 596 | 1 004 | 311 | 30.98 (30.96, 31.00) | 81 127 | 5 115 | 380 | 7.43 (7.42, 7.43) | 80 723 | 5 436 | 1 624 | 29.87 (29.87, 29.88) | 79 255 | 6 297 | 1100 | 17.47 (17.46, 17.48) |  |
|  | 5 (most deprived) | 81 575 | 1 317 | 421 | 31.97 (31.95, 31.98) | 80 945 | 5 678 | 502 | 8.84 (8.84, 8.85) | 80 417 | 6 024 | 2 027 | 33.65 (33.64, 33.66) | 78 657 | 6 506 | 1469 | 22.58 (22.57, 22.59) |  |
| **Home ownership** | Own outright (REF) | 215 694 | 2 187 | 560 | 25.61 (25.59, 25.62) | 214 648 | 13 395 | 640 | 4.78 (4.78, 4.78) | 213 516 | 13 416 | 2 761 | 20.58 (20.58, 20.58) | 210 782 | 16 211 | 1 687 | 10.41 (10.40, 10.41) |  |
|  | Own with mortgage | 155 193 | 1 669 | 501 | 30.02 (30.00, 30.03) | 154 542 | 8 195 | 788 | 9.62 (9.61, 9.62) | 153 916 | 10 281 | 3 665 | 35.65 (35.64, 35.65) | 150 827 | 11 528 | 2 333 | 20.24 (20.23, 20.24) |  |
|  | Rent - LA/council/housing association | 2 4031 | 567 | 175 | 30.86 (30.84, 30.89) | 23 760 | 2 114 | 126 | 5.96 (5.95, 5.97) | 2 3623 | 2 021 | 623 | 30.83 (30.81, 30.84) | 2 3041 | 2 184 | 497 | 22.76 (22.74, 22.77) |  |
|  | Rent - private landlord | 13 827 | 198 | 65 | 32.83 (32.78, 32.88) | 13 737 | 878 | 74 | 8.43 (8.42, 8.44) | 13 669 | 966 | 322 | 33.33 (33.31, 33.35) | 13 390 | 1 085 | 240 | 22.12 (22.10, 22.14) |  |
|  | Shared ownership | 1 288 | 23 | 9 | 39.13 (38.99, 39.27) | 1 278 | 75 | 9 | 12.00 (11.95, 12.05) | 1 270 | 101 | 35 | 34.65 (34.59, 34.72) | 1241 | 116 | 23 | 19.83 (19.78, 19.88) |  |
|  | Live rent free | 3 074 | 29 | 11 | 37.93 (37.80, 38.06) | 3 056 | 208 | 14 | 6.73 (6.71, 6.76) | 3038 | 208 | 68 | 32.69 (32.65, 32.74) | 2978 | 237 | 46 | 19.41 (19.37, 19.45) |  |

Test + = Number of positive tests

## Supplementary Table 3: Test positivity for test-independent risk factors

| Risk factor | Level of exposure | Time 1 | | | | Time 2 | | | | Time 3 | | | | Time 4 | | | |
| --- | --- | --- | --- | --- | --- | --- | --- | --- | --- | --- | --- | --- | --- | --- | --- | --- | --- |
|  |  | Total sample | Tests | Test + | Test positivity % (95% CI) | Total sample | Tests | Test + | Test positivity % (95% CI) | Total sample | Tests | Test + | Test positivity % (95% CI) | Total sample | Tests | Test + | Test positivity % (95% CI) |
| Total | | 420 231 | 4 805 | 1 353 | 28.16 (28.15, 28.17) | 418 087 | 25 382 | 1 678 | 6.61 (6.61, 6.61) | 416 055 | 27 536 | 7 613 | 27.65 (27.64, 27.65) | 409 150 | 31 964 | 4 930 | 15.42 (15.42, 15.43) |
| Blood type | A | 178 251 | 2 067 | 584 | 28.25 (28.24, 28.27) | 177 339 | 10 812 | 715 | 6.61 (6.61, 6.62) | 176 454 | 11 860 | 3 377 | 28.47 (28.47, 28.48) | 173 357 | 13 533 | 2 080 | 15.37 (15.37, 15.37) |
|  | B | 38 919 | 493 | 141 | 28.60 (28.57, 28.63) | 38 708 | 2 411 | 172 | 7.13 (7.13, 7.14) | 38 518 | 2 548 | 731 | 28.69 (28.68, 28.70) | 37 871 | 2 914 | 479 | 16.44 (16.43, 16.45) |
|  | AB | 14 812 | 152 | 50 | 32.89 (32.84, 32.95) | 14 740 | 891 | 65 | 7.30 (7.28, 7.31) | 14 665 | 969 | 294 | 30.34 (30.32, 30.36) | 14 403 | 1 147 | 204 | 17.79 (17.77, 17.80) |
|  | O | 175 773 | 1 891 | 521 | 27.55 (27.54, 27.57) | 174 913 | 10 478 | 684 | 6.53 (6.52, 6.53) | 174 068 | 11 244 | 2 964 | 26.36 (26.35, 26.37) | 171 402 | 13 423 | 2 012 | 14.99 (14.98, 14.99) |
| Hair colour | Blonde | 44 478 | 487 | 128 | 26.28 (26.26, 26.31) | 44 270 | 2 648 | 160 | 6.04 (6.04, 6.05) | 44 060 | 2 928 | 803 | 27.42 (27.41, 27.44) | 43 318 | 3 523 | 544 | 15.44 (15.43, 15.45) |
|  | Brown | 316 498 | 3 452 | 933 | 27.03 (27.02, 27.04) | 314 975 | 18 945 | 1 256 | 6.63 (6.63, 6.63) | 313 381 | 20 473 | 5 562 | 27.17 (27.16, 27.17) | 308 365 | 23 744 | 3 430 | 14.45 (14.44, 14.45) |
|  | Other | 57 455 | 842 | 284 | 33.73 (33.71, 33.75) | 57 059 | 3 655 | 256 | 7.00 (7.00, 7.01) | 56 837 | 4 017 | 1 210 | 30.12 (30.11, 30.13) | 55 729 | 4 544 | 918 | 20.20 (20.19, 20.21) |

Test + = Number of positive tests

## Supplementary Table 4: Association between test-associated risk factors and SARs-CoV-2 testing

| Risk factor | Level of exposure | Whole year  OR (95% CI) | Time 1  OR (95% CI) | Time 2  OR (95% CI) | Time 3  OR (95% CI) | Time 4  OR (95% CI) |
| --- | --- | --- | --- | --- | --- | --- |
| Education | Degree or higher | REF | | | | |
|  | Vocational qualifications | 1.18 (1.15, 1.2) | 1.21 (1.12, 1.31) | 1.17 (1.13, 1.21) | 1.22 (1.18, 1.26) | 1.13 (1.1, 1.17) |
|  | AS/A level | 1.05 (1.01, 1.09) | 0.97 (0.83, 1.12) | 1.08 (1.01, 1.14) | 1.03 (0.97, 1.1) | 1.04 (0.99, 1.1) |
|  | GCSE or less | 1.22 (1.2, 1.25) | 1.25 (1.16, 1.35) | 1.24 (1.2, 1.28) | 1.27 (1.23, 1.32) | 1.15 (1.12, 1.19) |
| Income | Greater than £100,000 | REF | | | | |
|  | £52,000-£100,000 | 0.95 (0.91, 0.99) | 0.99 (0.83, 1.16) | 0.91 (0.85, 0.98) | 0.94 (0.88, 1) | 0.95 (0.89, 1.01) |
|  | £31,000-51,599 | 1.01 (0.97, 1.05) | 1.03 (0.87, 1.2) | 0.96 (0.89, 1.02) | 1.05 (0.98, 1.11) | 0.99 (0.94, 1.05) |
|  | £18,000-£30,999 | 1.02 (0.98, 1.06) | 1.15 (0.96, 1.34) | 0.99 (0.93, 1.06) | 1.07 (1, 1.14) | 0.99 (0.93, 1.04) |
|  | Less than £18,000 | 1.16 (1.11, 1.21) | 1.63 (1.36, 1.89) | 1.23 (1.14, 1.31) | 1.18 (1.1, 1.26) | 1.08 (1.01, 1.14) |
| Accommodation type | House or bungalow | REF | | | | |
|  | Flat, maisonette or apartment | 1.07 (1.03, 1.1) | 1.51 (1.37, 1.65) | 1.21 (1.16, 1.27) | 1.03 (0.99, 1.08) | 1.05 (1.01, 1.1) |
|  | Mobile or temporary structure (e.g., caravan) | 0.99 (0.76, 1.21) | 1.51 (0.38, 2.65) | 1.12 (0.74, 1.5) | 1.06 (0.69, 1.43) | 0.98 (0.66, 1.3) |
|  | Sheltered accommodation | 1.61 (1.38, 1.85) | 3.62 (2.48, 4.75) | 1.88 (1.5, 2.25) | 1.3 (1.01, 1.6) | 1.64 (1.32, 1.96) |
|  | Care home | 3.04 (1.97, 4.69) | 4.77 (2.2, 10.35) | 2.82 (1.61, 4.95) | 3.26 (1.93, 5.51) | 1.91 (0.38, 3.44) |
|  | | | | | | |
| IMD Quintile | 1 (least deprived) | REF | | | | |
|  | 2 | 1.00 (0.97, 1.02) | 1.08 (0.98, 1.20) | 1.02 (0.98, 1.06) | 1.01 (0.97, 1.05) | 0.96 (0.92, 1.00) |
|  | 3 | 1.04 (1.01, 1.06) | 1.17 (1.05, 1.29) | 1.05 (1.01, 1.10) | 1.05 (1.01, 1.10) | 1.02 (0.98, 1.06) |
|  | 4 | 1.09 (1.06, 1.12) | 1.42 (1.29, 1.56) | 1.16 (1.11, 1.21) | 1.10 (1.06, 1.14) | 1.04 (1.00, 1.08) |
|  | 5 (most deprived) | 1.22 (1.19, 1.25) | 1.88 (1.71, 2.06) | 1.33 (1.28, 1.39) | 1.24 (1.19, 1.29) | 1.09 (1.05, 1.13) |
| Home ownership | Own outright | REF | | | | |
|  | Own with mortgage | 1.13 (1.10, 1.15) | 1.27 (1.18, 1.37) | 1.10 (1.06, 1.13) | 1.17 (1.14, 1.21) | 1.09 (1.05, 1.12) |
|  | Rent - LA/council/housing association | 1.47 (1.42, 1.52) | 2.58 (2.34, 2.84) | 1.71 (1.63, 1.79) | 1.47 (1.40, 1.54) | 1.32 (1.26, 1.39) |
|  | Rent - private landlord | 1.18 (1.13, 1.24) | 1.59 (1.37, 1.85) | 1.27 (1.18, 1.37) | 1.22 (1.13, 1.30) | 1.13 (1.06, 1.21) |
|  | Shared ownership | 1.29 (1.13, 1.48) | 1.99 (1.32, 3.02) | 1.16 (0.91, 1.46) | 1.38 (1.12, 1.69) | 1.33 (1.09, 1.61) |
|  | Live rent free | 1.14 (1.04, 1.25) | 1.03 (0.72, 1.50) | 1.29 (1.12, 1.49) | 1.16 (1.01, 1.34) | 1.10 (0.96, 1.25) |

## Supplementary Table 5: Association between test-independent risk factors and SARs-CoV-2 testing

| Risk factor | Level of exposure | Whole year  OR (95% CI) | Time 1  OR (95% CI) | Time 2  OR (95% CI) | Time 3  OR (95% CI) | Time 4  OR (95% CI) |
| --- | --- | --- | --- | --- | --- | --- |
| Blood type | A | REF | | | | |
|  | B | 1 (0.97, 1.03) | 1.04 (0.94, 1.15) | 1.02 (0.98, 1.07) | 0.97 (0.92, 1.01) | 0.98 (0.94, 1.02) |
|  | AB | 0.98 (0.93, 1.02) | 0.87 (0.72, 1.01) | 1 (0.93, 1.07) | 0.97 (0.91, 1.04) | 1.02 (0.95, 1.08) |
|  | O | 0.97 (0.95, 0.99) | 0.91 (0.85, 0.97) | 0.98 (0.95, 1.01) | 0.95 (0.93, 0.98) | 1 (0.98, 1.03) |
| Hair colour | Blonde | REF | | | | |
|  | Brown | 0.98 (0.95, 1) | 1.01 (0.91, 1.1) | 1.01 (0.97, 1.05) | 0.98 (0.94, 1.02) | 0.94 (0.9, 0.97) |
|  | Other | 1.03 (1, 1.07) | 1.19 (1.05, 1.33) | 1.06 (1.01, 1.12) | 1.04 (0.98, 1.09) | 0.98 (0.93, 1.02) |

## Supplementary Table 6: Association between test-associated risk factors and testing positive for SARs-CoV-2 infection

| Risk factor | Level of exposure | Whole year  OR (95% CI) | Time 1  OR (95% CI) | Time 2  OR (95% CI) | Time 3  OR (95% CI) | Time 4  OR (95% CI) |
| --- | --- | --- | --- | --- | --- | --- |
| Education | Degree or higher | REF | | | | |
|  | Vocational qualifications | 1.37 (1.3, 1.43) | 1.38 (1.14, 1.62) | 1.14 (0.98, 1.3) | 1.27 (1.17, 1.36) | 1.47 (1.35, 1.6) |
|  | AS/A level | 1.11 (1.01, 1.21) | 0.96 (0.63, 1.3) | 0.93 (0.67, 1.18) | 1.15 (0.99, 1.31) | 1.23 (1.04, 1.42) |
|  | GCSE or less | 1.55 (1.48, 1.63) | 1.25 (1.04, 1.47) | 1.35 (1.17, 1.54) | 1.5 (1.39, 1.61) | 1.69 (1.55, 1.83) |
| Income | Greater than £100,000 | REF | | | | |
|  | £52,000-£100,000 | 1.17 (1.05, 1.29) | 1.45 (0.86, 2.04) | 0.96 (0.7, 1.22) | 1.15 (0.98, 1.32) | 1.29 (1.06, 1.52) |
|  | £31,000-51,599 | 1.4 (1.26, 1.53) | 1.65 (0.99, 2.31) | 0.95 (0.7, 1.21) | 1.23 (1.05, 1.41) | 1.8 (1.49, 2.11) |
|  | £18,000-£30,999 | 1.44 (1.3, 1.59) | 1.63 (0.98, 2.29) | 0.9 (0.65, 1.15) | 1.27 (1.09, 1.46) | 1.87 (1.54, 2.2) |
|  | Less than £18,000 | 1.6 (1.44, 1.77) | 1.77 (1.06, 2.47) | 0.97 (0.7, 1.24) | 1.29 (1.1, 1.49) | 2.2 (1.8, 2.59) |
| Accommodation type | House or bungalow | REF | | | | |
|  | Flat, maisonette or apartment | 1.03 (0.96, 1.1) | 1.07 (0.85, 1.29) | 0.89 (0.71, 1.07) | 0.9 (0.81, 1) | 1.1 (0.98, 1.23) |
|  | Mobile or temporary structure (e.g. caravan) | 0.76 (0.26, 1.25) | NA | NA | 0.94 (0.11, 1.78) | 0.93 (-0.07, 1.93) |
|  | Sheltered accomodation | 1.31 (0.9, 1.72) | 0.9 (0.27, 1.54) | 0.88 (0.06, 1.7) | 1.24 (0.6, 1.88) | 1.32 (0.63, 2.02) |
|  | Care home | 1.75 (0.9, 3.39) | NA | 2.45 (0.76, 7.91) | 1.19 (0.49, 2.88) | 7.0 (2.68, 18.27) |
|  | | | | | | |
| IMD Quintile | 1 (least deprived) | REF | | | | |
|  | 2 | 1.15 (1.08, 1.23) | 0.88 (0.67, 1.09) | 1.06 (0.84, 1.28) | 1.2 (1.08, 1.32) | 1.17 (1.03, 1.31) |
|  | 3 | 1.25 (1.17, 1.34) | 0.81 (0.61, 1) | 1.2 (0.96, 1.43) | 1.3 (1.17, 1.43) | 1.27 (1.12, 1.41) |
|  | 4 | 1.44 (1.34, 1.53) | 1.2 (0.93, 1.46) | 1.33 (1.08, 1.59) | 1.35 (1.22, 1.48) | 1.58 (1.41, 1.76) |
|  | 5 (most deprived) | 1.66 (1.55, 1.76) | 1.22 (0.95, 1.48) | 1.39 (1.13, 1.64) | 1.47 (1.33, 1.62) | 1.97 (1.76, 2.19) |
| Home ownership | Own outright | REF | | | | |
|  | Own with mortgage | 1.33 (1.27, 1.39) | 1.21 (1, 1.42) | 1.14 (0.99, 1.3) | 1.3 (1.21, 1.39) | 1.31 (1.21, 1.42) |
|  | Rent - LA/council/housing assoc | 1.47 (1.37, 1.58) | 1.33 (1.04, 1.61) | 0.89 (0.7, 1.07) | 1.22 (1.09, 1.36) | 1.74 (1.53, 1.95) |
|  | Rent - private landlord | 1.43 (1.29, 1.57) | 1.41 (0.95, 1.88) | 1.2 (0.86, 1.54) | 1.27 (1.07, 1.46) | 1.55 (1.3, 1.8) |
|  | Shared ownership | 1.61 (1.16, 2.06) | 1.69 (0.18, 3.2) | 2.1 (0.54, 3.66) | 1.32 (0.75, 1.89) | 1.43 (0.75, 2.1) |
|  | Live rent free | 1.38 (1.1, 1.67) | 1.91 (0.44, 3.38) | 1.12 (0.47, 1.76) | 1.34 (0.92, 1.76) | 1.56 (1.04, 2.09) |

## Supplementary Table 7: Association between test-independent risk factors and testing positive for SARs-CoV-2 infection

| Risk factor | Level of exposure | Whole year  OR (95% CI) | Time 1  OR (95% CI) | Time 2  OR (95% CI) | Time 3  OR (95% CI) | Time 4  OR (95% CI) |
| --- | --- | --- | --- | --- | --- | --- |
| Blood type | A | REF | | | | |
|  | B | 0.97 (0.91, 1.04) | 0.93 (0.72, 1.14) | 1.08 (0.88, 1.27) | 0.97 (0.87, 1.07) | 0.97 (0.86, 1.09) |
|  | AB | 1.11 (1, 1.22) | 1.25 (0.8, 1.7) | 1.17 (0.84, 1.49) | 1.04 (0.89, 1.2) | 1.16 (0.97, 1.35) |
|  | O | 0.91 (0.87, 0.95) | 0.93 (0.8, 1.06) | 0.98 (0.87, 1.09) | 0.88 (0.83, 0.93) | 0.94 (0.88, 1.01) |
| Hair colour | Blonde | REF | | | | |
|  | Brown | 0.95 (0.9, 1.01) | 1.01 (0.79, 1.23) | 1.12 (0.92, 1.32) | 0.98 (0.89, 1.07) | 0.9 (0.81, 0.99) |
|  | Other | 1.08 (1, 1.16) | 1.09 (0.8, 1.39) | 1.14 (0.89, 1.4) | 1.02 (0.9, 1.14) | 1.14 (0.99, 1.28) |

## Supplementary Table 8: Association between test-associated risk factors and testing negative for SARs-CoV-2 infection

| Risk factor | Level of exposure | Whole year  OR (95% CI) | Time 1  OR (95% CI) | Time 2  OR (95% CI) | Time 3  OR (95% CI) | Time 4  OR (95% CI) |
| --- | --- | --- | --- | --- | --- | --- |
| Education | Degree or higher | REF | | | | |
|  | Vocational qualifications | 1.11 (1.09, 1.14) | 1.11 (1.01, 1.21) | 1.16 (1.12, 1.2) | 1.13 (1.09, 1.18) | 1.07 (1.03, 1.1) |
|  | AS/A level | 1.03 (0.99, 1.08) | 0.98 (0.81, 1.15) | 1.08 (1.01, 1.15) | 1 (0.93, 1.07) | 1.02 (0.96, 1.08) |
|  | GCSE or less | 1.14 (1.11, 1.16) | 1.19 (1.08, 1.29) | 1.21 (1.17, 1.26) | 1.15 (1.11, 1.19) | 1.07 (1.04, 1.1) |
| Income | Greater than £100,000 | REF | | | | |
|  | £52,000-£100,000 | 0.91 (0.87, 0.95) | 0.92 (0.74, 1.09) | 0.92 (0.85, 0.98) | 0.89 (0.83, 0.96) | 0.92 (0.86, 0.98) |
|  | £31,000-51,599 | 0.94 (0.9, 0.98) | 0.93 (0.76, 1.1) | 0.96 (0.89, 1.03) | 0.98 (0.91, 1.05) | 0.91 (0.85, 0.97) |
|  | £18,000-£30,999 | 0.94 (0.9, 0.99) | 1.03 (0.84, 1.22) | 1 (0.93, 1.07) | 0.98 (0.91, 1.06) | 0.89 (0.84, 0.95) |
|  | Less than £18,000 | 1.07 (1.02, 1.12) | 1.43 (1.16, 1.69) | 1.23 (1.14, 1.32) | 1.09 (1.01, 1.17) | 0.96 (0.89, 1.02) |
| Accommodation type | House or bungalow | REF | | | | |
|  | Flat, maisonette or apartment | 1.09 (1.05, 1.12) | 1.49 (1.32, 1.65) | 1.23 (1.17, 1.29) | 1.08 (1.02, 1.14) | 1.04 (0.99, 1.09) |
|  | Mobile or temporary structure (e.g. caravan) | 0.99 (0.76, 1.23) | 2.02 (0.51, 3.54) | 1.15 (0.76, 1.55) | 1.04 (0.64, 1.44) | 0.99 (0.65, 1.33) |
|  | Sheltered accommodation | 1.62 (1.37, 1.87) | 3.67 (2.34, 5.01) | 1.88 (1.5, 2.26) | 1.25 (0.93, 1.58) | 1.58 (1.24, 1.92) |
|  | Care home | 2.97 (1.86, 4.73) | 6.8 (3.14, 14.74) | 2.68 (1.48, 4.84) | 3.34 (1.85, 6.03) | 1 .00 (0.46, 2.18) |
|  | | | | | | |
| IMD Quintile | 1 (least deprived) | REF | | | | |
|  | 2 | 1 (0.97, 1.03) | 1.1 (0.97, 1.24) | 1.06 (1.01, 1.1) | 0.99 (0.94, 1.03) | 0.96 (0.92, 1) |
|  | 3 | 1.04 (1.02, 1.07) | 1.22 (1.07, 1.36) | 1.1 (1.05, 1.15) | 1.03 (0.98, 1.08) | 1.03 (0.99, 1.07) |
|  | 4 | 1.07 (1.04, 1.1) | 1.28 (1.13, 1.43) | 1.19 (1.14, 1.24) | 1.05 (1, 1.1) | 1.01 (0.97, 1.05) |
|  | 5 (most deprived) | 1.17 (1.13, 1.2) | 1.63 (1.44, 1.82) | 1.37 (1.31, 1.43) | 1.15 (1.09, 1.2) | 1.02 (0.98, 1.06) |
| Home ownership | Own outright | REF | | | | |
|  | Own with mortgage | 1.06 (1.04, 1.09) | 1.2 (1.1, 1.31) | 1.07 (1.03, 1.1) | 1.08 (1.04, 1.12) | 1.04 (1.01, 1.08) |
|  | Rent - LA/council/housing assoc | 1.39 (1.34, 1.44) | 2.25 (1.99, 2.51) | 1.69 (1.6, 1.78) | 1.36 (1.28, 1.44) | 1.21 (1.14, 1.27) |
|  | Rent - private landlord | 1.12 (1.07, 1.18) | 1.47 (1.19, 1.74) | 1.25 (1.15, 1.35) | 1.12 (1.02, 1.22) | 1.04 (0.96, 1.12) |
|  | Shared ownership | 1.19 (1.01, 1.38) | 1.6 (0.75, 2.45) | 1.07 (0.8, 1.33) | 1.22 (0.92, 1.53) | 1.28 (1.01, 1.55) |
|  | Live rent free | 1.05 (0.94, 1.16) | 0.89 (0.48, 1.31) | 1.25 (1.07, 1.44) | 1.02 (0.84, 1.2) | 1.02 (0.87, 1.17) |

## Supplementary Table 9: Association between test-independent risk factors and testing negative for SARs-CoV-2 infection

| Risk factor | Level of exposure | Whole year  OR (95% CI) | Time 1  OR (95% CI) | Time 2  OR (95% CI) | Time 3  OR (95% CI) | Time 4  OR (95% CI) |
| --- | --- | --- | --- | --- | --- | --- |
| Blood type | A | REF | | | | |
|  | B | 1 (0.97, 1.03) | 1.06 (0.93, 1.18) | 1.02 (0.97, 1.07) | 0.98 (0.93, 1.03) | 0.98 (0.93, 1.02) |
|  | AB | 0.97 (0.92, 1.01) | 0.82 (0.66, 0.99) | 0.99 (0.92, 1.07) | 0.96 (0.88, 1.04) | 1 (0.93, 1.07) |
|  | O | 0.99 (0.97, 1.01) | 0.92 (0.85, 0.99) | 0.98 (0.96, 1.01) | 0.99 (0.95, 1.02) | 1.01 (0.98, 1.04) |
| Hair colour | Blonde | REF | | | | |
|  | Brown | 0.98 (0.96, 1.01) | 1.01 (0.9, 1.12) | 1 (0.96, 1.05) | 0.99 (0.94, 1.04) | 0.95 (0.91, 0.99) |
|  | Other | 1.02 (0.98, 1.05) | 1.15 (0.99, 1.32) | 1.06 (1, 1.12) | 1.03 (0.97, 1.1) | 0.95 (0.9, 1) |

## Supplementary Table 10: Association between age at study commencement and sex with receiving a test for SARs-CoV-2, testing positive for SARs-CoV-2 infection and testing negative for SARs-CoV-2 infection

| Risk factor | Outcome | Level of exposure | Whole year | Time 1 | Time 2 | Time 3 | Time 4 |
| --- | --- | --- | --- | --- | --- | --- | --- |
| Age | Testing | ≤40 | REF | | | | |
|  |  | >40 & ≤45 | 0.95 (0.87, 1.03) | 1.13 (0.79, 1.46) | 1.14 (0.96, 1.33) | 1.01 (0.88, 1.14) | 0.86 (0.76, 0.96) |
|  |  | >45 & ≤50 | 0.94 (0.86, 1.02) | 0.94 (0.66, 1.22) | 1.18 (0.99, 1.37) | 0.96 (0.84, 1.08) | 0.87 (0.77, 0.97) |
|  |  | >50 & ≤55 | 0.9 (0.82, 0.97) | 0.77 (0.54, 1.01) | 1.22 (1.02, 1.42) | 0.88 (0.77, 1) | 0.84 (0.74, 0.94) |
|  |  | >55 & ≤60 | 0.9 (0.83, 0.98) | 0.73 (0.51, 0.95) | 1.37 (1.15, 1.59) | 0.86 (0.75, 0.97) | 0.84 (0.74, 0.94) |
|  |  | >60 & ≤65 | 1.02 (0.94, 1.11) | 0.93 (0.65, 1.2) | 1.68 (1.42, 1.95) | 0.96 (0.84, 1.08) | 0.93 (0.82, 1.04) |
|  |  | >65 | 1.28 (1.18, 1.39) | 1.53 (1.07, 1.98) | 2.26 (1.9, 2.62) | 1.21 (1.05, 1.36) | 1.07 (0.94, 1.19) |
|  | Testing positive | ≤40 | REF | | | | |
|  |  | >40 & ≤45 | 0.91 (0.76, 1.05) | 1.63 (0.49, 2.77) | 0.63 (0.39, 0.87) | 0.86 (0.65, 1.08) | 0.92 (0.69, 1.15) |
|  |  | >45 & ≤50 | 0.72 (0.61, 0.83) | 1.38 (0.42, 2.35) | 0.46 (0.28, 0.63) | 0.72 (0.54, 0.9) | 0.76 (0.57, 0.94) |
|  |  | >50 & ≤55 | 0.48 (0.4, 0.55) | 0.63 (0.39, 0.87) | 0.28 (0.17, 0.39) | 0.5 (0.38, 0.63) | 0.5 (0.38, 0.63) |
|  |  | >55 & ≤60 | 0.3 (0.25, 0.35) | 1.17 (0.35, 1.99) | 0.18 (0.11, 0.26) | 0.32 (0.24, 0.39) | 0.29 (0.22, 0.37) |
|  |  | >60 & ≤65 | 0.24 (0.2, 0.27) | 0.86 (0.65, 1.08) | 0.15 (0.09, 0.21) | 0.23 (0.17, 0.29) | 0.23 (0.17, 0.29) |
|  |  | >65 | 0.24 (0.2, 0.28) | 1.26 (0.39, 2.13) | 0.12 (0.08, 0.17) | 0.22 (0.16, 0.27) | 0.22 (0.16, 0.27) |
|  | Testing negative | ≤40 | REF | | | | |
|  |  | >40 & ≤45 | 0.99 (0.89, 1.09) | 0.98 (0.64, 1.32) | 1.25 (1.02, 1.47) | 1.08 (0.89, 1.28) | 0.88 (0.76, 1.01) |
|  |  | >45 & ≤50 | 1.05 (0.94, 1.16) | 0.86 (0.56, 1.16) | 1.34 (1.1, 1.59) | 1.11 (0.92, 1.31) | 0.94 (0.81, 1.07) |
|  |  | >50 & ≤55 | 1.11 (1, 1.23) | 1.25 (1.02, 1.47) | 1.46 (1.19, 1.72) | 1.17 (0.97, 1.38) | 0.99 (0.86, 1.13) |
|  |  | >55 & ≤60 | 1.23 (1.1, 1.35) | 0.7 (0.46, 0.94) | 1.68 (1.38, 1.98) | 1.29 (1.06, 1.52) | 1.08 (0.93, 1.22) |
|  |  | >60 & ≤65 | 1.45 (1.3, 1.59) | 1.08 (0.89, 1.28) | 2.08 (1.71, 2.46) | 1.55 (1.28, 1.82) | 1.22 (1.05, 1.38) |
|  |  | >65 | 1.82 (1.64, 2.01) | 1.44 (0.95, 1.93) | 2.82 (2.31, 3.32) | 1.96 (1.62, 2.31) | 1.4 (1.21, 1.6) |
| Sex | Testing | Male | REF | | | | |
|  |  | Female | 0.92 (0.91, 0.94) | 0.86 (0.81, 0.91) | 0.89 (0.86, 0.91) | 0.95 (0.93, 0.98) | 0.94 (0.91, 0.96) |
|  | Testing positive | Male | REF | | | | |
|  |  | Female | 0.96 (0.93, 1) | 0.8 (0.7, 0.9) | 0.92 (0.82, 1.01) | 0.94 (0.89, 0.99) | 1.04 (0.97, 1.1) |
|  | Testing negative | Male | REF | | | | |
|  |  | Female | 0.92 (0.91, 0.94) | 0.92 (0.86, 0.98) | 0.89 (0.87, 0.92) | 0.97 (0.94, 1) | 0.93 (0.91, 0.95) |

## Supplementary Table 11: Association between test-independent risk factors, ABO blood type and hair colour, with tests for SARs-CoV-2, testing positive for SARs-CoV-2 infection and testing negative for SARs-CoV-2 infection, excluding individuals of non-White British ancestry

| Risk factor | Outcome | Level of exposure | Whole year  OR (95% CI) | Time 1  OR (95% CI) | Time 2  OR (95% CI) | Time 3  OR (95% CI) | Time 4  OR (95% CI) |
| --- | --- | --- | --- | --- | --- | --- | --- |
| Blood type | Testing | A | REF | | | | |
|  |  | B | 0.99 (0.95, 1.02) | 1.03 (0.9, 1.15) | 1 (0.94, 1.05) | 0.95 (0.9, 1) | 0.98 (0.93, 1.03) |
|  |  | AB | 0.98 (0.93, 1.03) | 0.86 (0.69, 1.02) | 1.01 (0.93, 1.09) | 0.98 (0.9, 1.05) | 1 (0.93, 1.07) |
|  |  | O | 0.97 (0.95, 0.99) | 0.91 (0.85, 0.98) | 0.98 (0.95, 1.01) | 0.94 (0.91, 0.97) | 1 (0.97, 1.03) |
|  | Test positive | A | REF | | | | |
|  |  | B | 0.94 (0.87, 1.01) | 0.95 (0.69, 1.22) | 1.06 (0.84, 1.29) | 0.91 (0.8, 1.02) | 0.94 (0.81, 1.07) |
|  |  | AB | 1.11 (0.99, 1.24) | 1.31 (0.76, 1.87) | 1.02 (0.68, 1.36) | 1.05 (0.86, 1.23) | 1.2 (0.97, 1.43) |
|  |  | O | 0.91 (0.87, 0.95) | 0.97 (0.81, 1.12) | 0.97 (0.85, 1.09) | 0.88 (0.82, 0.94) | 0.97 (0.89, 1.04) |
|  | Test negative | A | REF | | | | |
|  |  | B | 0.99 (0.96, 1.03) | 1.03 (0.89, 1.17) | 0.99 (0.94, 1.05) | 0.98 (0.92, 1.04) | 0.98 (0.93, 1.03) |
|  |  | AB | 0.97 (0.92, 1.02) | 0.79 (0.6, 0.98) | 1.02 (0.93, 1.1) | 0.96 (0.88, 1.05) | 0.98 (0.9, 1.06) |
|  |  | O | 0.99 (0.97, 1.01) | 0.92 (0.84, 0.99) | 0.99 (0.96, 1.02) | 0.97 (0.94, 1.01) | 1.01 (0.98, 1.04) |
|  | | | | | | | |
| Hair colour | Testing | Blonde | REF | | | | |
|  |  | Brown | 0.97 (0.94, 1) | 0.99 (0.89, 1.09) | 1.01 (0.96, 1.05) | 0.97 (0.92, 1.01) | 0.93 (0.9, 0.97) |
|  |  | Other | 1.01 (0.98, 1.05) | 1.1 (0.95, 1.25) | 1.04 (0.98, 1.1) | 1.04 (0.98, 1.1) | 0.99 (0.94, 1.05) |
|  | Test positive | Blonde | REF | | | | |
|  |  | Brown | 0.95 (0.89, 1.01) | 1.05 (0.81, 1.29) | 1.07 (0.88, 1.27) | 0.98 (0.89, 1.08) | 0.9 (0.81, 1) |
|  |  | Other | 1 (0.91, 1.08) | 0.84 (0.58, 1.1) | 1.04 (0.78, 1.3) | 1.04 (0.78, 1.3) | 1.05 (0.9, 1.21) |
|  | Test negative | Blonde | REF | | | | |
|  |  | Brown | 0.98 (0.95, 1.01) | 0.99 (0.87, 1.1) | 1.01 (0.96, 1.05) | 0.97 (0.92, 1.02) | 0.95 (0.91, 0.99) |
|  |  | Other | 1.02 (0.97, 1.06) | 1.15 (0.97, 1.33) | 1.04 (0.98, 1.11) | 1.04 (0.98, 1.11) | 0.99 (0.93, 1.04) |

## Supplementary Table 12: Association between income and tests for SARs-CoV-2, with tests for SARs-CoV-2, testing positive for SARs-CoV-2 infection and testing negative for SARs-CoV-2 infection, excluding individuals who self-reported being retired at baseline

| Outcome | Income strata | Whole year | Time 1 | Time 2 | Time 3 | Time 4 |
| --- | --- | --- | --- | --- | --- | --- |
| Testing | Greater than £100,000 | REF | | | | |
|  | £52,000-£100,000 | 0.94 (0.9, 0.98) | 0.99 (0.82, 1.17) | 0.91 (0.84, 0.97) | 0.93 (0.87, 1) | 0.94 (0.88, 1) |
|  | £31,000-51,599 | 1 (0.96, 1.05) | 1.05 (0.87, 1.24) | 0.95 (0.88, 1.02) | 1.03 (0.96, 1.1) | 0.98 (0.92, 1.04) |
|  | £18,000-£30,999 | 1.03 (0.99, 1.08) | 1.18 (0.97, 1.39) | 0.99 (0.91, 1.07) | 1.09 (1.01, 1.16) | 0.98 (0.92, 1.05) |
|  | Less than £18,000 | 1.17 (1.11, 1.22) | 1.64 (1.34, 1.93) | 1.28 (1.18, 1.38) | 1.17 (1.09, 1.26) | 1.09 (1.01, 1.16) |
| Testing positive | Greater than £100,000 | REF | | | | |
|  | £52,000-£100,000 | 1.17 (1.05, 1.29) | 1.45 (0.83, 2.07) | 0.92 (0.66, 1.17) | 1.19 (1.01, 1.37) | 1.29 (1.05, 1.53) |
|  | £31,000-51,599 | 1.46 (1.31, 1.61) | 1.68 (0.97, 2.39) | 0.94 (0.68, 1.2) | 1.33 (1.13, 1.53) | 1.89 (1.55, 2.22) |
|  | £18,000-£30,999 | 1.55 (1.38, 1.71) | 1.63 (0.92, 2.34) | 0.9 (0.64, 1.15) | 1.45 (1.22, 1.67) | 1.96 (1.59, 2.32) |
|  | Less than £18,000 | 1.51 (1.35, 1.67) | 1.68 (0.95, 2.41) | 0.81 (0.57, 1.05) | 1.2 (1.01, 1.39) | 2.22 (1.8, 2.64) |
| Testing negative | Greater than £100,000 | REF | | | | |
|  | £52,000-£100,000 | 0.91 (0.86, 0.95) | 0.92 (0.73, 1.1) | 0.92 (0.85, 0.99) | 0.88 (0.81, 0.95) | 0.91 (0.85, 0.97) |
|  | £31,000-51,599 | 0.92 (0.88, 0.97) | 0.94 (0.75, 1.13) | 0.95 (0.88, 1.03) | 0.94 (0.87, 1.02) | 0.88 (0.82, 0.94) |
|  | £18,000-£30,999 | 0.94 (0.9, 0.99) | 1.05 (0.83, 1.26) | 1 (0.92, 1.08) | 0.97 (0.89, 1.05) | 0.88 (0.82, 0.94) |
|  | Less than £18,000 | 1.08 (1.03, 1.14) | 1.47 (1.17, 1.77) | 1.31 (1.2, 1.42) | 1.1 (1.01, 1.2) | 0.95 (0.88, 1.02) |
